# Comparison of Shear Bond Strength and Microleakage between Activa™ Bioactive Restorative™ and Bulk-Fill Composites—An In Vitro Study

**DOI:** 10.3390/polym15132840

**Published:** 2023-06-27

**Authors:** Laura Martínez-Sabio, Lissethe Peñate, María Arregui, Ana Veloso Duran, José Raúl Blanco, Francisco Guinot

**Affiliations:** 1 Pediatric Dentistry Department, Faculty of Dentistry, Universitat Internacional de Catalunya, 08195 Sant Cugat del Vallès, Spain; lauramsabio@uic.es (L.M.-S.); aveloso@uic.es (A.V.D.); fguinot@uic.es (F.G.); 2 Restorative Dentistry Department, Faculty of Dentistry, Universitat Internacional de Catalunya, 08195 Sant Cugat del Vallès, Spain; lissethe@uic.es; 3 Restorative Department, Faculty of Dentistry, Interamerican University of Panama, Panama City 081604737, Panama; jrblancoc@hotmail.com

**Keywords:** shear bond strength, microleakage, dental composite, bulk-fill composite, active bioactive

## Abstract

Bioactive materials have emerged as a promising alternative to conventional restorative materials as part of more conservative dentistry. The aim of this study was to evaluate and compare the shear bond strength (SBS) and microleakage of a new bioactive restorative material, two bulk-fill restorative composites, and a conventional composite at 24 h, 4 weeks, and 8 weeks. Three hundred and sixty molars and premolars were divided into four groups: ACTIVA™ BioACTIVE Restorative™, Filtek™ Bulk-Fill Restorative Composite, Tetric^®^ N-Ceram Bulk-Fill Composite, and G-aenial^®^ Composite. The normality of the data was determined with the Kolmogorov-Smirnov test, then the two-way ANOVA and Fisher’s test were used for analyzing SBS data, and the Kruskal-Wallis and DSCF tests were conducted to analyze the microleakage. In the SBS test, there were no statistically significant differences between materials (*p* = 0.587), and the relation between material and time (*p* = 0.467), time points showed statistically significant differences (*p* = 0.016). As for the microleakage, statistically significant differences were found for all three time periods (*p* < 0.05), showing the conventional composite to have the lowest microleakage, followed by the bioactive material, and lastly the two bulk-fill composites. In conclusion, the new bioactive material has similar evaluated properties to bulk-fill composites (bond strength) and conventional composites (bond strength and microleakage) and can be used as an alternative restorative material.

## 1. Introduction

The evolution of dentistry is closely related to the advances and development of dental materials. Dentists have been continuously searching for ideal restorative dental materials [1]. Conventional restorative materials have been associated with a large number of limitations. For example, amalgam is related to less conservative cavity preparations, is not suitable for aesthetic restorations, and has an environmental impact [2]. While the evolution of resin-based materials has addressed these concerns, it has also brought with it some new challenges, such as postoperative tooth hypersensitivity, polymerization shrinkage, and microleakage [2,3,4]. Except for dental composites, glass-ionomer cements (GICs) have been used as an aesthetic material in low-stress concentration areas because of their chemical bonding and anticariogenic properties. However, they lack physical and aesthetic properties compared to composites [5].

Bulk-fill composites were introduced to simplify the application protocol of conventional composites with the incremental technique, which diminishes the polymerization shrinkage but is time-consuming and may lead to air entrapment between consecutive layers of the composite resin [6]. The main difference with conventional composites is that they have an increased depth of cure as a result of their increased translucency, which improves light penetration [7,8]. This greater depth of cure allows the placement of 4–5 mm-thick increments, which shortens the time of the clinical procedure and facilitates the application protocol [8]. Bulk-fill composites have been reported to promote less polymerization shrinkage stress than conventional microhybrid composites performed by the incremental filling technique [6].

Over the past two decades, most of the research has focused on the development of bioactive materials. These have emerged as a promising alternative to conventional materials, and they play a key role in the progress towards more conservative dentistry [1,9]. A bioactive material is defined as a material that has the effect of eliciting a response from living tissue, organisms, or cells, such as promoting the formation of hydroxyapatite [10]. Therefore, this new generation of bioactive materials introduces the opportunity to induce the repair and regeneration of lost dental hard tissues by mimicking the physiological mechanisms of mineralization [9]. Presently, this type of material is a good treatment option for primary teeth, especially in class I and class II preparation of primary molars [11].

ACTIVA™ BioACTIVE Restorative™ (Pulpdent, USA) has been recently introduced to the market as a hybridization between a resin-modified glass ionomer cement (RMGI) and a resin composite. The manufacturer claims that this is the first bioactive dental material with an ionic resin matrix, a shock-absorbing resin component, and bioactive fillers that mimic the physical and chemical properties of natural teeth [12,13]. The bioactive properties of ACTIVA™ products are based on a mechanism in which the material responds to pH cycles and plays an active role in the release and recharge of calcium, phosphate, and fluoride ions [14,15]. It is assumed that they perform chemical bonding to the tooth structure, which leads to the sealing of the cavity margins, thereby minimizing gap formation at the tooth-restoration interface and microleakage [13,16].

ACTIVA™ BioACTIVE Restorative™ is delivered via a twin-cylinder automix syringe and is theoretically a true bulk-fill material [17], which can be applied with or without a bonding agent. However, according to the manufacturer, it is advisable to use a bonding agent in cases of additional retention [18]. Previous clinical and in vitro studies showed a higher level of failure when the material was used without the application of a bonding agent [19]. The material has three setting mechanisms: it cures with low-intensity light for 20 s per layer and has both glass-ionomer (acid-base reaction) and composite self-cure setting reactions. It is recommendable to allow the material to self-cure for 15–20 s before light curing [20,21]. In addition, ACTIVA™ products do not contain bisphenol A (BPA), bisphenol A glycidyl methacrylate (bis-GMA), or other BPA derivatives, which are associated with polymerization shrinkage and stress [4,22].

There is continuous research towards the development of an ideal restorative material as well as new adhesives to reduce microleakage and improve bond strength. Nevertheless, despite the advances in these materials, marginal integrity remains a challenge, being the major feature of current adhesive restorative materials [18]. As a newly introduced material, ACTIVA™ literature is very scarce, and some of the few available studies are controversial [4,22]. Hence, the present study aimed to evaluate and compare shear bond strength and microleakage between bulk-fill and conventional composites and ACTIVA™ BioACTIVE Restorative™ at 24 h, 4 weeks, and 8 weeks. The null hypotheses of this study were: (1) there was no difference in the bond strength of the ACTIVA™ BioACTIVE Restorative™ material at 24 h, 4 weeks, and 8 weeks compared to other restorative materials; and (2) there was no difference in the microleakage of the ACTIVA™ BioACTIVE Restorative™ material at 24 h, 4 weeks, and 8 weeks compared to other restorative materials.

## 2. Materials and Methods

This in vitro study was conducted with the approval of the University Ethics Research Committee in June 2021 (ODP-ELM-2021-01) at the Universitat Internacional de Catalunya (Barcelona, Spain) between July 2021 and March 2022.

For the sample size calculation, the Granmo calculator was used (version 7.12, Municipal Institute for Medical Research, Barcelona, Spain). For both SBS and microleakage, the minimum sample size was calculated to be 13 in each group, assuming an alfa risk of 0.05, considering that it is the probability of rejecting the null hypothesis when it is true, and a beta risk of 0.2, taking into account that it is the probability of accepting the null hypothesis when it is false, with a standard deviation of 0.4 and a size effect of 0.63 between the variables SBS-time and microleakage-time. As the dropout rate was not estimated in the sample size determination, it was decided that the final sampling rate would be 15 specimens per group.

One hundred and eighty extracted molars (maxillary and mandibular) and one hundred and eighty extracted premolars (maxillary and mandibular) were each divided into four groups: ACTIVA™ BioACTIVE Restorative™, Filtek™ Bulk-Fill Restorative Composite, Tetric^®^ N-Ceram Bulk-Fill Composite, and G-aenial^®^ Composite (*n* = 45). Molars were used for shear bond strength testing because they have more dentine surface for area bonding, and premolars were used for Class II preparations for micro-leakage testing, as they have been used in other studies in the literature [4,22]. Teeth with restorations or structural defects that would compromise the procedure were excluded. The teeth were cleaned with a saline solution and then stored in a 10% formalin-neutral buffered solution after extraction to maintain hydration. Table 1 shows the composition of restorative materials used in this study.

### 2.1. Shear Bond Strength

#### 2.1.1. Specimen Preparation

Each molar was transversely sectioned in the middle of the crown with a double-sided diamond blade under constant irrigation at low speed (Isomet 1000, Buehler, Lake Bluff, IL, USA) to obtain a flat dentin surface. Then, the molars were embedded in acrylic resin perpendicular to the base. Any residual resin that could cover the dentin surface was polished with grit sandpaper (P.320; Hiltech Europe MP Series, Corsico, Italy) at 50 rpm for 60 s under constant water running.

An etch and rinse protocol was used following the manufacturer’s recommendations. Phosphoric acid 37% (Total Etch, Ivoclar Vivadent, Liechtenstein—Batch number Z01KSS) was used for 15 s and then rinsed with water for 15 s. The teeth were dried with oil-free air for 5 s. A microbrush was used to scrub the dentin with a universal bonding agent, Prime and Bond Active^®^ (Dentsply Sirona, Konstanz, Germany—Batch number 2102000523), for 20 s, and the solvent was left to evaporate for 5 s. Finally, each specimen was polymerized for 20 s using a light-emitting diode (LED) unit (Demi™, SDS Kerr Corp, Orange, CA, USA.) with an intensity of 1200 mW/cm^2^.

**Figure 1:**
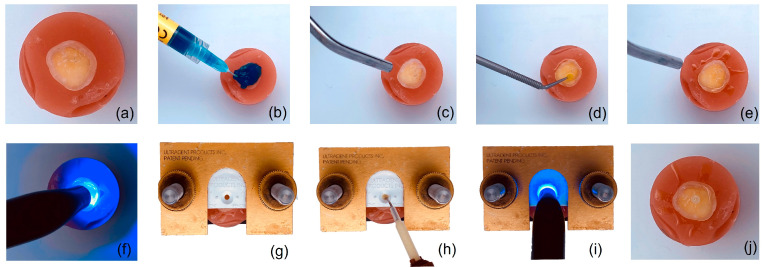
Step-by-step procedure for specimen preparation for SBS test. (**a**) Specimen embedded in acrylic resin. (**b**) Etching. (**c**) Rinsing and drying. (**d**) Bonding. (**e**) Drying. (**f**) Polymerization. (**g**) Specimen in clamp. (**h**) Application of restorative material. (**i**) Polymerization. (**j**) Specimen prepared.

Then the acrylic cylinders were positioned into the bonding clamp and the restorative material was condensed across the bonding mold insert until it was filled, the dimensions were a diameter of 2 ± 0.4 mm and a length of 3 ± 0.2 mm, and at the end all the excess was removed (Figure 1). After polymerization, the screws of the bonding clamp were released, and the bond between the restorative material and the tooth was confirmed. The teeth were then stored in distilled water at 37 °C ± 1 °C for 24 h (*n* = 60), 4 weeks (*n* = 60), and 8 weeks (*n* = 60) before the SBS test.

#### 2.1.2. Shear Bond Strength Protocol

For the shear bond strength (SBS) test, the specimens were placed in the test base clamp and positioned in the Ultra Tester Bond Strength testing machine (Ultradent Products S.A., South Jordan, UT, USA). A 2 mm-thick knife-edge blade was used to apply a vertical loading force with a load cell of 50N max. and an accuracy class of 0.1% at a crosshead speed of 1 mm/min until failure of the bond between resin-based composite and tooth. The shear bond strength was noted in MPa.

#### 2.1.3. Fracture Type Evaluation

After specimens were tested, fracture type was evaluated with a stereomicroscope (1.5× magnifications) (Discovery V8, Zeiss, Oberkochen, Germany) and classified as adhesive fracture, cohesive fracture, and mixed fracture. A cohesive failure occurs when the fracture occurs completely within the dentin structure or within the restorative material; an adhesive failure occurs when the bond fails at the interface of the adhesive and the restorative material; and it is considered mixed when a combination of adhesive and cohesive fractures occurs [4].

### 2.2. Microleakage

#### 2.2.1. Specimen and Cavity Preparation

The premolars were embedded in gypsum perpendicular to the base.

**Figure 2:**
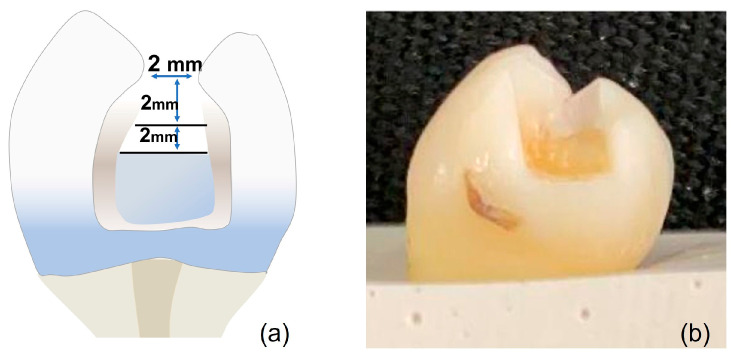
Design of the cavity for microleakage group. (**a**) Diagram with the measurements of the preparation; (**b**) Cavity made for the study.

The cavity design was class II restorations with 2 × 2 × 2 mm +/− 0.5 measurements prepared using a carbide bur #245 (Komet Dental, Lemgo, Germany) and measured with a periodontal probe to maintain standardization of the cavities (Figure 2).

#### 2.2.2. Restorative Procedure

The etch and rinse protocol was used following the manufacturer’s recommendations. Phosphoric acid 37% (Total Etch, Ivoclar Vivadent, Liechtenstein) was used for 15 s and then rinsed with water for 15 s. The teeth were dried with oil-free air for 5 s. A microbrush was used to scrub the dentin with a universal bonding agent, Prime and Bond Active^®^ (Dentsply Sirona, Konstanz, Germany), for 20 s, and the solvent was left to evaporate for 5 s. Finally, each specimen was polymerized for 20 s using a light-emitting diode (LED) unit (DemiTM, SDS Kerr Corp.) with an intensity of 1200 mW/cm^2^.

After the bonding protocol was complete, a metal matrix was adapted, and the restorative material was placed. The ACTIVA™ BioACTIVE Restorative™ was injected with a metal tip cannula; bulk-fill composites were condensed in 4 mm increments and the conventional composite in 2 mm increments. Each increment of material was polymerized for 20 s. In the case of ACTIVA™ BioACTIVE Restorative™, prior to the polymerization, the material was let to sit for 20–30 s for self-curing, as the manufacturer recommended, as it is a dual-cure material. Once the restoration was finished, the excess material was removed and polished with paper discs.

#### 2.2.3. Microleakage Stain Protocol

Teeth were covered with a dark nail varnish except for 1 mm from the restoration. Each group was stored in different reservoirs with 1% methylene blue dye for 24 h (*n* = 60), 4 weeks (*n* = 60), and 8 weeks (*n* = 60). Then, the teeth were cleaned with tap water until the excess methylene blue was removed.

Teeth were sectioned longitudinally with a double-sided diamond blade under constant irrigation at low speed (Isomet 1000, Buehler, Germany) to obtain two sections from each tooth.

#### 2.2.4. Microleakage Evaluation

**Figure 3:**
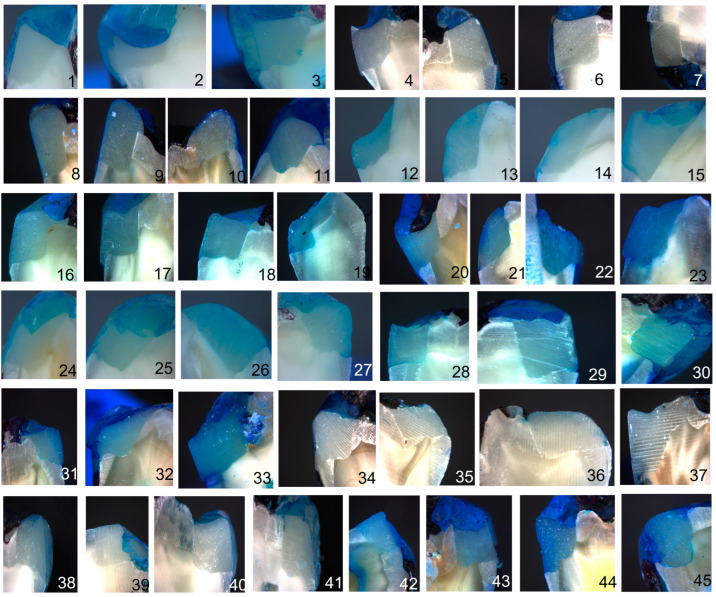
Microleakage of the different materials evaluated at each time: (1) ACTIVA^TM^ 24 h. score 0; (2) ACTIVA^TM^ 24 h. score 1; (3) ACTIVA^TM^ 24 h. score 2; (4) ACTIVA^TM^ 4 weeks score 0; (5) ACTIVA^TM^ 4 weeks score 1; (6) ACTIVA^TM^ 4 weeks score 2; (7) ACTIVA^TM^ 4 weeks score 3; (8) ACTIVA^TM^ 8 weeks score 0; (9) ACTIVA^TM^ 8 weeks score 1; (10) ACTIVA^TM^ 8 weeks score 2; (11) ACTIVA^TM^ 8 weeks score 3; (12) Tetric^®^ N-Ceram 24 h. score 0; (13) Tetric^®^ N-Ceram 24 h. score 1; (14) Tetric^®^ N-Ceram 24 h. score 2; (15) Tetric^®^ N-Ceram 24 h. score 3; (16) Tetric^®^ N-Ceram 4 weeks score 0; (17) Tetric^®^ N-Ceram 4 weeks score 1; (18) Tetric^®^ N-Ceram 4 weeks score 2; (19) Tetric^®^ N-Ceram 4 weeks score 3; (20) Tetric^®^ N-Ceram 8 weeks score 0; (21) Tetric^®^ N-Ceram 8 weeks score 1; (22) Tetric^®^ N-Ceram 8 weeks score 2; (23) Tetric^®^ N-Ceram 8 weeks score 3; (24) Filtek^TM^ 24 h. score 0; (25) Filtek^TM^ 24 h. score 1; (26) Filtek^TM^ 24 h. score 2; (27) Filtek^TM^ 24 h. score 3; (28) Filtek^TM^ 4 weeks score 0; (29) Filtek^TM^ 4 weeks score 2; (30) Filtek^TM^ 4 weeks score 3; (31) Filtek^TM^ 8 weeks score 1; (32) Filtek^TM^ 8 weeks score 2; (33) Filtek^TM^ 8 weeks score 3; (34) G-aenial^®^ 24 h. score 0; (35) G-aenial^®^ 24 hscore 1 ; (36) G-aenial^®^ 24 h. score 2; (37) G-aenial^®^ 24 h. score 3; (38) G-aenial^®^ 4 weeks score 0; (39) G-aenial^®^ 4 weeks score 1; (40) G-aenial^®^ 4 weeks score 2; (41) G-aenial^®^ 4 weeks score 3; (42) G-aenial^®^ 8 weeks score 0; (43) G-aenial^®^ 8 weeks score 1; (44) G-aenial^®^ 8 weeks score 2; (45) G-aenial^®^ 8 weeks score 3.

Dye penetration extent was evaluated with a stereomicroscope (1.0× magnifications) (Discovery V8, Zeiss, Germany) and scored between 0 and 3 for each side of every tooth. Score 0 was attributed to the absence of dye penetration; score 1 was attributed to stain at the interface; score 2 was attributed to stain across the interface < 1 mm; and score 3 was attributed to stain across the interface ≥ 1 mm. The highest score for each tooth was the one used (Figure 3).

### 2.3. Statistical Analysis

The statistical analysis was carried out using the Jamovi statistical program (The jamovi project (2021). Jamovi (version 2.2.5) [Computer software]. Retrieved from https://www.jamovi.org). For SBS results, the Kolmogorov-Smirnov test was performed to check the normal distribution of data, the two-way ANOVA to analyze the adhesion results, and the Fisher’s exact test for the type of failure. For microleakage results, data was analyzed with the Kruskal-Wallis test to determine the statistical significance of the comparison within groups over time and subsequently with the DSCF test for peer comparisons. Regarding microleakage, the leakage area was measured to determine whether the leakage was between material-adhesive or adhesive-tooth; the Chi-square test was performed for this purpose. In addition, a Pearson correlation was conducted between microleakage and leakage area at the three study times and between the materials and each time point. Additionally, a Pearson correlation analysis was performed to establish whether there was a correlation between SBS and microleakage as a function of time. A confidence level of 95% was used in all tests, and a significant difference was accepted at *p* < 0.05.

## 3. Results

### 3.1. SBS Results

The comparison between groups at three time points was performed with a two-way ANOVA, and there were no statistical differences between materials (*p* = 0.587) or between materials and times (*p* = 0.467). The only group that presented statistically significant differences was time (*p* = 0.016). Subsequently, the Fisher’s post-hoc test of study time group was performed, and it was observed that statistically significant differences were found between study times of 24 h and 4 weeks (*p* = 0.005) and between 4 and 8 weeks (*p* = 0.05); no statistically significant differences were observed between 24 h and 8 weeks (*p* = 0.393).

In Table 2, it can be appreciated that the material with the highest bond strength is different for each time point and that the bond values for all materials are higher at 4 weeks than at 24 h or 8 weeks, except for ACTIVA™, whose highest score is at 8 weeks. In all groups, the values remain within the range considered ideal (20–30 MPa). The conventional composite (G-aenial) is the only one that has less SBS at 8 weeks than 24 h.

**Figure 4:**
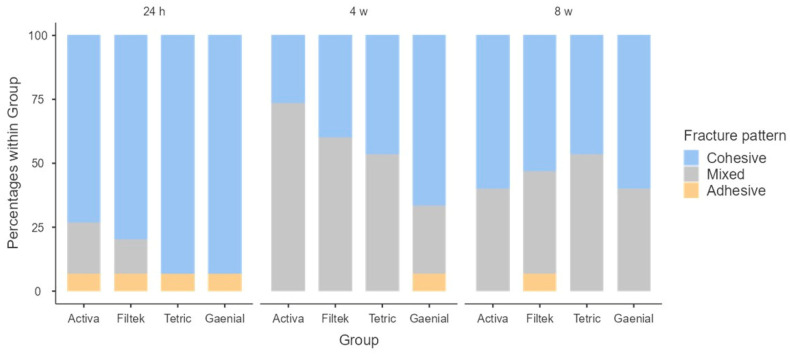
Fracture pattern frequency of the different materials at 24 h, 4 weeks, and 8 weeks.

Figure 4 shows that the most frequent type of failure was the cohesive type, both at 24 h and at 8 weeks. The materials with the highest rates of this type of fracture were the control composite (93.3% at 24 h and 60% at 8 weeks) and the Bulk Fill composites: at 24 h, Tetric^®^ N-Ceram Bulk Fill was 93.3%, and at 8 weeks, Filtek™ Bulk Fill was 53.3%. At 4 weeks, it was observed that the most frequent type of failure in all materials was the mixed fracture pattern (ACTIVA™ 73.3%, Filtek™ Bulk Fill 60%, and Tetric^®^ N-Ceram Bulk Fill 53.3%), except for the control composite with 66.7% cohesive failure. Fisher’s test showed that there are no statistically significant differences between the different materials at the different study times: 24 h (*p* = 0.429), 4 weeks (*p* = 0.121), and 8 weeks (*p* = 0.897).

### 3.2. Microleakage Results

To analyze the microleakage data, the non-parametric Kruskall-Wallis test was performed, and it was observed that the three time groups showed statistically significant differences: 24 h (*p* = 0.007), 4 weeks (*p* < 0.001), and 8 weeks (*p* = 0.045).

In the pairwise comparison, it was observed that at 24 h, the ACTIVA™ group showed statistically significant differences with the two Bulk-Fill materials, Filtek™ Bulk Fill Composite (*p* = 0.021) and Tetric^®^ N-Ceram Bulk Fill (*p* = 0.048), but not with the conventional composite (*p* = 0.986). In contrast, at 4 weeks, statistically significant differences were observed between ACTIVA™ and Filtek™ Bulk-Fill (*p* = 0.017) and also between both types of Bulk-Fill composites and the conventional composite (G-aenial), in this case Filtek™ Bulk Fill composite (*p* = 0.006) and Tetric^®^ N-Ceram Bulk Fill (*p* = 0.026). Whereas, in the 8-week group, no statistically significant differences (*p* > 0.05) were observed between the different materials in the pairwise comparison.

**Figure 5:**
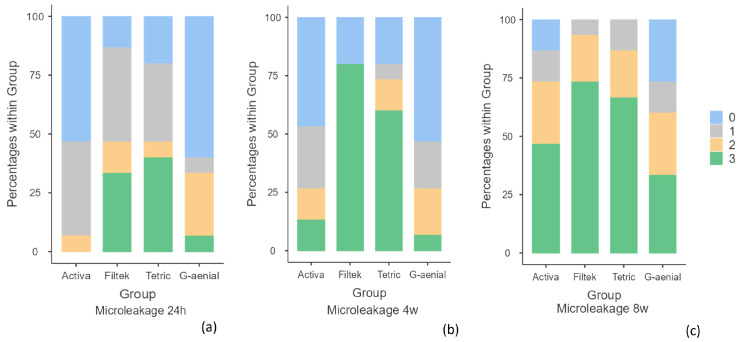
Microleakage frequency at each time point: (**a**) 24 h; (**b**) 4 weeks; (**c**) 8 weeks. Legend score 0: absence of dye penetration; 1: stain at the interface; 2: stain across the interface < 1 mm; 3: stain across the interface ≥ 1 mm.

In Table 3, it can be appreciated that the material with the lowest level of microleakage in the three time periods was the conventional composite with no dye penetration, followed by ACTIVA™ BioACTIVE Restorative™. On the other hand, the materials with the highest microleakage were Bulk-Fill composites. It was observed that at 24 h, Tetric^®^ EvoCeram Bulk-Fill showed a 40% microleakage score of 3 and Filtek™ Bulk-Fill 33.3%; at 4 weeks, Filtek™ had 80% and Tetric^®^ 60%, and at 8 weeks, Filtek™ had 73.3% and Tetric^®^ 66.7% (Figure 5).

The chi-square test was conducted to analyze the leakage, and statistically significant differences were observed at 24 h (*p* = 0.009), while no statistically significant differences were observed at 4 weeks (*p* = 0.215) or 8 weeks (*p* = 0.156). Table 4 shows that at the three time points, the highest leakage rate in all materials was between material and adhesive. At 24 h and 4 weeks, the high non-leakage rates can be seen in Activa and G-aenial.

A Pearson correlation was performed between both variables of microleakage and leakage area as a function of time. The correlation was positive at all three time points, which implies that there is a strong relationship between variables, particularly at 24 h and 4 weeks, and decreases slightly at 8 weeks. Similarly, statistically significant differences were obtained at the three time points, as can be seen in Table 5.

According to the materials and time, all the groups showed a positive correlation except Tetric at 8 weeks, which was negative with no statistically significant differences between the two variables, as shown in Table 6.

### 3.3. Correlation between SBS and Microleakage

The correlation between the three study times between SBS and microleakage was negative, which implies that there was no relationship between the two variables. Furthermore, there were no statistically significant differences at any of the three time periods. Table 7 shows the correlation and *p* values between SBS and microleakage.

## 4. Discussion

Presently, restorative dentistry creates new materials that require fewer application steps, thus reducing the risk of contamination and treatment time [23]. In addition, these materials must be able to provide good marginal integrity to achieve successful restorations. This in vitro study evaluated the bond strength and microleakage of a bioactive restorative material vs. a hybrid composite and two bulk-fill composites at 24 h, 4 weeks, and 8 weeks. The shear bond strength results showed no statistically significant differences; therefore, the first null hypothesis was accepted. However, microleakage differences at 24 h and 4 weeks were observed among the different materials evaluated, but they were not observed after 8 weeks. Therefore, the second null hypothesis was partially rejected.

Even though the shear bond strength method has been used in the literature for evaluating adhesive strength at the interface among dental restorative materials, adhesive, and the substrate [24,25], because its application is relatively simple and reproducible compared to other methods in which the steps to align the specimen inside the machine are more difficult without affecting the stress distribution [26], it has been observed that there is a great influence between different parameters that affect both the materials studied, the area of adhesion, the storage conditions, and the design of the test.

Taking into account that configurations of the applied forces or the type of shear/cut that is used are parameters of great variability [27], it would be useful to analyze the behavior of the materials used in the present study subjected to different tests such as micro shear bond strength (µSBS), macro-tensile bond strength (TBS), micro-tensile bond strength test (µTBS), mold-enclosed shear bond strength (ME-SBS/EM-SBS), or lever-induced mold-enclosed shear bond strength (LIME-SBS) tests [28,29] as future research. Therefore, despite the shear bond strength test being disputable and considered not ideal, and with plenty of variables needing to be held in consideration, the preference for conventional shear tests is justified because they are easy to perform, requiring minimal equipment and specimen preparation, and provide an overview of the adhesion strength; that is why, presently, they are still extensively used for adhesion evaluation of dental materials [30,31]. Researchers must always ensure that the methodology used is sufficiently accurate and standardized. A simple bond test, such as the shear bond strength test (SBS), provides a first indication of bond performance but is complemented by several tests in independent laboratories, which are more discriminatory than actual and should also be complemented by a variety of complementary testing protocols. Alternative testing protocols using different approaches, such as micro-tension, micro-shear, static or dynamic fatigue, and fracture toughness testing, do not necessarily yield different data, but in most cases of the adhesive effect, it is worth noting that similar comparable results are obtained for the more traditional test, the shear bond strength protocol [32].

In the assessment of bonding effectiveness, researchers should consider improving the standardization of laboratory study variables for the bond strength tests, following the guidelines of the International Organization for Standardization (ISO). This will help to ensure reliable and consistent results across different studies [33]. Despite little standardization on the bond strength methods and different variables reported in the literature, the mean value of the dentin bond strength measured in a lot of studies was 12.97 ± 6.29 MPa [34]. The results of the present study about SBS were within the range from 20 to 30 MPa, according to authors like Pashley et al. [35], who suggested maintaining long-term values above 20 MPa. In this case, the minimum bond strength desired in adhesive materials was obtained.

The current results showed no statistically significant differences in the shear bond strength values of composite, Bulk-fill, and ACTIVA™ BioACTIVE-Restorative™ groups, which is in agreement with the findings of Tohidkhah et al. [4], despite the fact that their study conducted micro-tensile bond strength (μTBS) and the aging of their specimens was carried out during 3000 cycles of thermal cycling, corresponding to 16 weeks. Regarding the type of fracture, the present study did not find differences among groups. However, they found the mixed fracture to be the most frequent with ACTIVA™ BioACTIVE-Restorative™ and the cohesive fracture with the bulk-fill composites and the conventional composite. This difference could probably be related to the difference in methodology in the aging process and the fact that the maximum time of the present study was 8 weeks and its simulation was twice as long. On the other hand, SBS tests have been criticized for non-homogeneous stress distributions at the bonded interface, inducing cohesive failures within the substrates, which could also explain the prevalence of cohesive fractures in our results [31,36,37].

Other studies, such as Rifai et al. [22], are consistent with the present results. In their study, ACTIVA™ BioACTIVE-Restorative™ was used as an etch and rinse adhesive system, and the composite showed no statistically significant differences in SBS values. Therefore, comparing the results obtained in the different studies, the use of the adhesive system improves the results of the SBS values.

The results of this study, in terms of adhesion and microleakage, could be related to the materials’ composition. All the composites studied, except Tetric^®^ N-Ceram Bilk Fill, do not contain Bis-GMA; they are mainly composed of urethane dimethacrylate (UDMA), among other monomers. Literature has shown that UDMA exhibits superior mechanical properties to Bis-GMA, and an increase in shear strength has been observed [22].

An additional factor influencing the results obtained in this study could be related to the studied adhesive. Prime and Bond Active is considered a universal adhesive because it contains 10-methacryloyloxydecyl dihydrogen phosphate (10-MDP), a monomer that allows a chemical bond to the dental structure; it furthermore distinguishes itself as a HEMA- and TEGDMA-free adhesive. It has been observed that these two components can enhance the hydrolytic degradation of the materials due to their increased tendency to water sorption [38,39].

Due to all these characteristics of the main composition of UDMA-based composites and the absence of TEGDMA (both in adhesive and composites) and HEMA (in adhesive) that reduce the tendency to water sorption, it could be assumed that the adhesion and microleakage values obtained in this study are relatively stable for 8 weeks because of a possible slowdown in the hydrolytic degradation process for their composition. However, longer-term studies would be necessary to confirm these findings.

Another important factor of using a HEMA-free adhesive is that it allows the 10-MDP to have a stronger chemical bond with the dental structure, since this monomer in the presence of excess water reduces its adhesion strength [24,38,39].

All these factors related to the materials’ composition could be related to the high cohesive and mixed fracture rates obtained at all time points; although at 8 weeks they decreased, they were still higher than in other studies [19,22].

Related to the other parameter evaluated in this study, microleakage is considered a measure to evaluate the restoration performance at the marginal seal [16,24]. The dye penetration rate indicates the gaps between the tooth and the restorative material, which can allow the access of bacteria and their products [18]. Regarding microleakage, reports in the literature show that all fillings, irrespective of the restorative material used, show some degree of microleakage [18,23,40], which might be explained by polymerization shrinkage, cavity configuration, the difference in coefficient of thermal expansion, and other factors of each material. This agrees with the findings of the present study, in which the degree of dye penetration increased over time in all specimens. Nevertheless, the application and layering technique of resin-based composites influence the internal stress of direct restorations. Different layering approaches have been proposed to reduce internal stress, thus influencing bond strength and marginal adaptation [41].

In the pairwise comparison, there were no statistically significant differences found between ACTIVA™ BioACTIVE-Restorative™ and composite. As reported by other authors, such as Tohidkhah et al. [4], the use of adhesive with ACTIVA™ BioACTIVE-Restorative™ decreases microleakage at the enamel and dentin margins. Similar to the present study, Tohidkhah et al. [4] and Kaushik et al. [16] used universal adhesives, which contain 10-MDP, that will be relatively hydrolysis stable as water will be at a distance; this monomer is able to form strong ionic bonds with calcium (Ca) as the resultant Ca salt dissolves at a low dissolution rate in its own solution [42]. Omidi et al. [23] obtained similar results in their study in which they evaluated ACTIVA™ BioACTIVE-Restorative™ with and without conditioning; however, that study was conducted on primary teeth, so its results are not fully comparable with the results of the present study. Concerning the microleakage results between ACTIVA™ BioACTIVE-Restorative™ and bulk-fill composites, in this study statistically significant differences were observed in the first 24 h and 4 weeks, in which bulk-fill composites had a higher degree of dye penetration. However, at 8 weeks, there were no differences, which is in agreement with the results of Tohidkhah et al. [4] in the groups that have received surface treatment with adhesive, because the ACTIVA™ BioACTIVE-Restorative™ group without surface treatment had the highest degree of microleakage. A thorough assessment of the ACTIVA™ BioACTIVE-Restorative™ effectiveness may be limited by the in vitro research models. A more accurate representation of how the material could function in vivo could be achieved by developing a model that promotes a setting that permits ion exchange between the material and the tooth.

In addition, microleakage may be related to the composition of the materials studied. Kaushik et al. [18] justify that the lower microleakage observed in ACTIVA™ BioACTIVE-Restorative™ might be associated with the ability of the material to chemically bond with dental structure. The literature suggests that the acid phosphate groups have antibacterial properties that enhance the interaction between the resin and the bioactive glass particles that create a strong bond between the resin and hydroxyapatite, which results in a possible stronger seal that prevents microleakage [18,22].

In the case of G-aenial, a possible cause for the low microleakage observed is that it contains different types of pre-polymerized particles (strontium and lanthanoid fluoride) that contribute to reducing the shrinkage of the composite [43].

On the other hand, Bulk fill composites that have been specifically formulated to have a lower shrinkage and therefore less microleakage in this study are the materials that obtained the worst results at this point. In this case, the explanation of these results could be related more to the application technique than to the composition of the materials. As described by Cayo-Rojas et al. [44], in class II cavities, there is not enough guarantee that all the light reaches the bottom of the cavity properly due to the effect of the metal matrix. It has been observed that the matrix absorbs part of the emitted photons, thereby compromising the ability to properly polymerize all the photoinitiators.

This light scattering factor could be related to the result of obtaining a higher leakage area between the adhesive and the composite at all the times and materials studied.

Despite the literature proving that the effect of water on materials is a time-dependent variable [45], the results of this study show that in the present study the correlation between the variables and time is negative, so this relationship is not observed. It is possible that the immersion time is not long enough to observe the effect of time; longer-term studies should be done; or it could be related to the composition of materials because they have less water sorption than other materials.

Concerning the correlation between microleakage and leakage area, it is observed that the correlation at 8 weeks, although positive, is closer to 0 in all the groups, which could lead us to suspect that the effect of hydrolytic degradation could begin to have an effect. This is especially evident in Tetric, which has a negative correlation, which could be related to the fact that it is the only composite studied that has Bis-GMA in its composition, which causes a more evident tendency to water sorption than the other materials and therefore shows more evidence of hydrolytic degradation than the other composites.

Among the limitations of this study has been a lack of literature regarding ACTIVA™ BioACTIVE, which makes comparing the results to others difficult. On the one hand, microleakage studies are often used to compare bonding qualities, and while they are relatively easy to perform, the clinical significance of these tests and outcomes remains vague. On the other hand, the microleakage assessment method used in this study is based on a single longitudinal section, which results in irreversible loss of information because the specimen is partially discarded by sectioning. Moreover, only a limited number of points can be evaluated, which may lead to an underestimation or overestimation of the total leakage and to ignoring detailed information about the distribution pattern. It would be recommended to use a 3D microleakage assessment method to avoid these inconveniences in future investigations. Another limitation related to the microleakage is the use of dyes without considering the size of the bacteria or whether the dye could be representative or not of a clinical translation of the results. For that reason, this is an important topic for further research. Additionally, the short assessment period could lead to preliminary results that could change over time, and, of course, the in vitro aspect does not truly represent the oral conditions the materials are subjected to. To achieve more reliable results, studies with a longer assessment period are needed in order to assess the bioactive properties of ACTIVA™ BioACTIVE Restorative™.

## 5. Conclusions

Within the limitations of this in vitro study, it can be concluded that no statistically significant differences were found between the ACTIVA™ BioACTIVE Restorative™, the two bulk-fill composites, and the conventional composite regarding neither the shear bond strength nor the type of fracture at 24 h, 4 weeks, or 8 weeks.

As for the microleakage, statistically significant differences were found between the groups at all times studied, with the conventional composite being the one with lesser microleakage, followed by the bioactive material, and finally the two bulk-fill composites.

The new bioactive material has similar evaluated properties to bulk-fill composites (bond strength) and conventional composites (bond strength and microleakage) and can be used as an alternative restorative material.

## Figures and Tables

**Table 1:** Materials, composition, and batch number.

Material	Manufacturer	Batch Number	Composition
ACTIVA^TM^ BioACTIVE Restorative^TM^	Pulpdent, USA	210714	Powder: diurethane dimethacrylate, bis (2-(methacryloyloxy) ethyl). Phosphate, barium glass, ionomer glass, sodium fluoride, and colorants. Liquid: polyacrylic acid/maleic acid copolymer 160209.
Filtek^TM^ Bulk-Fill Restorative Composite	3M ESPE, USA	NC86129	Monomer matrix: aromatic urethane dimethacrylate (AUDMA), Addition-Fragmentation monomer (AFM), 1,12-dodecane-DMA, and urethane dimethacrylate (UDMA). Fillers: a combination of a non-agglomerated/non-aggregated 20-nm silica filler, a non-agglomerated/non-aggregated 4–11-nm zirconia filler, an aggregated zirconia/silica cluster filler (consisting of 20-nm silica and 4–11-nm zirconia particles), and an ytterbium trifluoride filler consisting of agglomerate 100-nm particles. The inorganic filler loading is about 76.5% by weight (58.4% by volume).
Tetric^®^ N-Ceram Bulk-Fill Composite	Ivoclar-Vivadent, Germany	Z01SWN	Monomer matrix: Bis-GMA, Bis-EMA, and UDMA; light initiator (Ivocerin^®^). Fillers: barium aluminium silicate glass with two different mean particle sizes; isofillers comprised of cured dimethacrylates, ytterbium fluoride, and spherical mixed oxide. The inorganic filler loading is about 75% by weight (61% by volume) and 17% polymer fillers, or Isofillers.
G-aenial^®^ Composite	GC, Japan	210420A	Monomer matrix: UDMA, dimethacrylate co-monomers (Bis-GMA free). Fillers: fluoroalumniosilicate, fumed silica, pre-polymerized fillers (silica, strontium, and lanthanoid fluoride), pigments, and catalysts (CQ and amine).

**Table 2:** Results of SBS group, mean ± standard deviation.

	Studied Time Points		
Material Group	24 h	4 Weeks	8 Weeks
ACTIVA™	22.00 ± 9.09	24.35 ± 7.06	25.35 ± 6.28
Filtek™	23.44 ± 10.43	26.43 ± 8.37	25.32 ± 5.13
G-aenial	22.03 ± 7.41	25.28 ± 6.08	20.67 ± 9.41
Tetric	20.93 ± 10.67	29.66 ± 8.79	22.29 ± 9.57

**Table 3:** Results of microleakage group.

Studied Time Points and Microleakage Score Rates												
Material Group	24 h				4 Weeks				8 Weeks			
	0	1	2	3	0	1	2	3	0	1	2	3
ACTIVA™	53.3%	40.0%	6.7%	0.0%	46.7%	26.7%	13.3%	13.3%	13.3%	13.3%	26.7%	46.7%
Filtek™	13.3%	40.0%	13.3%	33.3%	20.0%	0.0%	0.0%	80.0%	0.0%	6.7%	20.0%	73.3%
G-aenial	60.0%	6.7%	26.7%	6.7%	53.3%	20.0%	20.0%	6.7%	26.7%	13.3%	26.7%	33.3%
Tetric	20.0%	33.3%	6.7%	40.0%	20.0%	6.7%	13.3%	60.0%	0.0%	13.3%	20.0%	66.7%

Score 0 was attributed to the absence of dye penetration; score 1 was attributed to stain at the interface; score 2 was attributed to stain across the interface < 1 mm; and score 3 was attributed to stain across the interface ≥ 1 mm.

**Table 4:** Results of leakage area group.

Studied Time Points and Leakage Area Rates									
Material Group	24 h			4 Weeks			8 Weeks		
	N-L	T-A	M-A	N-L	T-A	M-A	N-L	T-A	M-A
ACTIVA™	60.0%	0.0%	40.0%	46.7%	13.3%	40.0%	20.0%	20.0%	60.0%
Filtek™	13.3%	6.7%	80.0%	20.0%	6.7%	73.3%	0.0%	26.7%	73.3%
G-aenial	60.0%	13.3%	26.7%	53.3%	0.0%	46.7%	26.7%	13.3%	60.0%
Tetric	20.0%	0.0%	80.0%	20.0%	6.7%	73.3%	26.7%	13.3%	60.0%

N-L: non-leakage; T-A: tooth-material; and M-A: material-adhesive.

**Table 5:** Correlation between microleakage and leakage as a function of time.

Time	Pearson (r)	*p* Value
24 h	0.743	<0.001
4 weeks	0.849	<0.001
8 weeks	0.452	<0.001

**Table 6:** Correlation between microleakage and leakage area in function of time and material.

Time						
Material Group	24 h		4 Weeks		8 Weeks	
	Pearson (r)	*p* Value	Pearson (r)	*p* Value	Pearson (r)	*p* Value
Activa	0.836	<0.001	0.806	<0.001	0.282	0.308
Filtek	0.473	0.075	0.952	<0.001	0.169	0.548
Tetric	0.699	0.004	0.820	<0.001	−0.255	0.359
G-aenial	0.880	<0.001	0.873	<0.001	0.686	0.005

**Table 7:** Correlation between variables as a function of time.

Time	Pearson (r)	*p* Value
24 h	−0.002	0.985
4 weeks	−0.090	0.492
8 weeks	−0.032	0.806

## Data Availability

The data presented in this study are available on request from the corresponding author.
